# Adult Pan-Colonic Intussusception Secondary to Poorly Differentiated Cecal Adenocarcinoma: A Rare Presentation of Stage IIA Colon Cancer

**DOI:** 10.7759/cureus.101382

**Published:** 2026-01-12

**Authors:** Caroline J Cushman, Mackenzie Coffin, Truman Archer, Muhammad H Nazim, Mohamed Elfedaly

**Affiliations:** 1 Department of Surgery, Texas Tech University Health Sciences Center, Amarillo, USA

**Keywords:** colectomy, colonic intussusception, colorectal carcinoma, intussusception, resection

## Abstract

Adult intussusception is rare, typically associated with an underlying pathology, and often presents with nonspecific symptoms. We report the case of a 68-year-old man presenting with diarrhea, abdominal pain, and weight loss. CT suggested volvulus versus intussusception, and emergent laparotomy revealed massive pan-colonic intussusception extending from the small bowel into the right colon and continuing through the transverse and descending colon to the sigmoid. A subtotal colectomy with end ileostomy was performed. The postoperative course was complicated by high serous drain output and transient hyponatremia, both managed conservatively. Pathology demonstrated a poorly differentiated cecal adenocarcinoma with weak neuroendocrine differentiation, staged pT3N0M0 (Stage IIA, American Joint Committee on Cancer 8th edition) with intact mismatch repair proteins. Oncology consultation identified high-risk features, and management options included adjuvant chemotherapy versus close observation with circulating tumor DNA and molecular profiling. This case depicts the rarity of adult pan-colonic intussusception, the need for oncologic resection given malignant lead points, and the importance of integrating molecular diagnostics into modern postoperative management.

## Introduction

Intussusception occurs when one segment of the bowel telescopes into another, leading to obstruction. It is predominantly a pediatric condition; adult cases represent 5% of all intussusceptions and only 1-5% of bowel obstructions [1,2]. Unlike pediatric intussusception, which is usually idiopathic, adult intussusception is pathological in up to 90% of cases, with malignancy accounting for over half [1,2]. Among the small number of adult cases of intussusception, most are limited to a short bowel segment; extensive pan-colonic intussusception is exceptionally rare. Indeed, very few case reports regarding pan-colonic intussusception have been reported. Because malignancy contributes to such a large proportion of adult intussusception cases, treatment often involves surgical resection and the need for a stoma. This case reports a massive adult intussusception involving nearly the entire colon, secondary to poorly differentiated cecal adenocarcinoma.

## Case presentation

**Figure 1:**
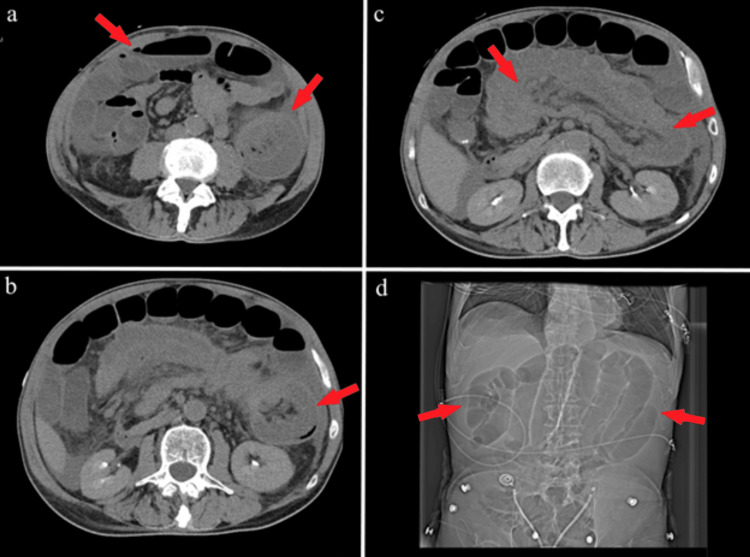
Preoperative CT imaging. (a) Left arrow: dilated bowel loops along with an air-fluid level concerning for small bowel obstruction; right arrow: mesenteric swirl raising the possibility of volvulus. (b) “Target” sign of bowel within bowel indicating possible intussusception. (c) Long section of mesenteric fat along a segment of intussusception. (d) Topogram showing multiple dilated loops of small bowel.

A 68-year-old man with no prior abdominal surgery presented with several weeks of diarrhea, worsening abdominal pain, and weight loss. Past medical history was notable for 30 pack-years of tobacco use. The patient reported no alcohol consumption, and family history was negative for colorectal malignancy. In the emergency room, vital signs were significant for a blood pressure of 109/90 mmHg. On physical examination, the patient's abdomen was greatly distended, tympanitic to percussion, and diffusely tender, and urgent imaging was performed. Initial CT of the abdomen/pelvis demonstrated mesenteric swirl concerning for volvulus versus intussusception (Figure 1).

Because of the high likelihood of an associated malignancy and bowel ischemia, as well as the possibility of mesenteric volvulus, the decision was made to proceed with an exploratory laparotomy. The patient underwent an emergent exploratory laparotomy, which revealed substantial intussusception with the small bowel telescoping into the right colon, extending through the transverse and descending colon to the sigmoid. Intraoperatively, the involved bowel demonstrated significant ischemia and compromised viability, precluding safe primary anastomosis or pouch reconstruction, and a subtotal colectomy with end ileostomy was performed without attempted reduction.

The patient’s postoperative course was marked by steady recovery with close monitoring of output and electrolytes. By postoperative day (POD) one, the ileostomy was functional, and the Jackson-Pratt (JP) drain produced serosanguineous output. On POD two, JP drainage peaked at 1.38 L/24 hours of serous fluid with a fluid creatinine of 0.7 mg/dL, consistent with non-urinous output; the diet was advanced to full liquids, and ostomy education was initiated. By POD three, the diet was further advanced to regular, the stoma appeared healthy, and antibiotics were discontinued. On POD four, JP output decreased to 620 mL/24 hours, though the patient developed mild hyponatremia (serum sodium 129 mmol/L), which was corrected with an intravenous saline bolus and oral salt supplementation. He was discharged home on POD five in stable condition with a functional ileostomy, JP drain in place producing 400-500 mL/day of serous fluid, and the wound VAC removed.

Histopathologic examination of the resected specimen revealed a poorly differentiated adenocarcinoma of the cecum measuring 3.2 × 2.2 cm with weak neuroendocrine differentiation. The tumor invaded through the muscularis propria into the pericolonic tissue. Resection margins were negative, and all 20 regional lymph nodes examined were free of tumor involvement. Tumor budding was intermediate (5-9), and no lymphovascular or perineural invasion was identified. Immunohistochemistry demonstrated intact mismatch repair proteins (MLH1, PMS2, MSH2, MSH6), indicating mismatch repair (MMR) stability, and the Ki-67 proliferation index was ≥90%. Based on the American Joint Committee on Cancer (AJCC) 8th edition, the tumor was staged as pT3N0M0, Stage IIA. The preoperative carcinoembryonic antigen level was 2.8 ng/mL.

At the two-week postoperative follow-up, the patient was evaluated by oncology, where the presence of high-risk features was noted. Adjuvant chemotherapy options, including capecitabine, 5-fluorouracil/leucovorin, or FOLFOX, were discussed in comparison with close observation guided by circulating tumor DNA (ctDNA) and microsatellite instability (MSI)/tumor mutational burden (TMB) profiling. At the four-week postoperative visit, he remained frail with a body mass index (BMI) of 16.1 kg/m² and an Eastern Cooperative Oncology Group performance status of 1. Laboratory evaluation demonstrated persistent iron-deficiency anemia (hemoglobin: 11.1 g/dL, ferritin: 11 ng/mL), for which oral iron supplementation was initiated.

At the four-week follow-up with oncology, the patient was ambulatory and able to keep up with light work. The decision was made to wait for additional study results before beginning adjuvant treatment. At the six-week follow-up with oncology, he remained anemic with a ferritin of 11 ng/mL and was advised to continue oral iron supplementation. However, ctDNA was still pending. At this visit, the patient agreed to begin a chemotherapy regimen of capecitabine 1,000 mg/m² (that is, 1,600 mg b.i.d.) for 14 days with cycles every 21 days. After being unable to reach by phone and missing several appointments, the patient was determined to be lost to follow-up.

## Discussion

Adult intussusception is an uncommon condition, accounting for approximately 5% of all intussusceptions and 1-5% of intestinal obstructions [1,2]. It is generally classified into four types: entero-enteric (confined to the small bowel), colo-colic (confined to the large bowel), ileocolic (terminal ileum prolapsing into the ascending colon), and ileocecal (with the ileocecal valve serving as the lead point) [1,2]. In children, most cases are idiopathic and benign, with hydrostatic reduction successful in up to 80% [1]. In contrast, intussusception in adults is a far more serious diagnosis: it is secondary and pathological in nearly 90% of presentations, with an identifiable lead point in the majority, most commonly a malignancy in colonic cases [1]. Reported etiologies include polyps, carcinoma, Meckel’s diverticulum, diverticula, and strictures [1,2]. The clinical presentation is often nonspecific, most commonly manifesting as features of bowel obstruction such as abdominal pain, nausea, vomiting, diarrhea, or weight loss. This frequently delays recognition and results in a diagnosis being made intraoperatively.

The extent of disease in this patient was particularly striking. Intraoperative findings revealed a pan-colonic intussusception extending from the small bowel into the sigmoid colon, an exceptionally rare phenomenon. Most reported cases describe segmental involvement limited to either the small bowel or a single colonic segment [1,3]. In current literature, total ileocolic intussusception, defined as small bowel telescoping through the entire colon to the rectum, is exceedingly rare. Frydman et al. (2013) reviewed the literature and identified only three prior adult cases (David et al. 2007; Chen et al. 2008; Ongom et al. 2013) in addition to their own, making four cases in total [4-7]. More recently, a case of incarcerated coloanal intussusception was described (McMahon et al., 2024), and with the present report, this represents approximately the sixth documented adult case of this extensive pattern (Table 1) [8].

**Table 1:** Reported cases of adult pan-colonic or total ileocolic intussusception (2007 to 2025). NR = not reported

Author/Year	Journal	Age/Sex	Presentation	Location/Extent	Operation	Lead point	Outcome/Follow-up
David et al. (2007) [5]	*Indian Journal of Enterology*	50, Male	Abdominal pain, prolapse	Ileum → rectum	Subtotal colectomy	None	Uneventful; follow-up NR
Chen et al. (2008) [6]	*Cases Journal*	36, Male	Abdominal pain, obstruction	Ileocecal → rectum	Subtotal colectomy	Ileocecal submucosal lipoma	Recovered; follow-up NR
Frydman et al. (2013) [4]	*World Journal of Emergency Surgery*	22, Female	Rectal prolapse, obstruction	Cecum → rectum	Right hemicolectomy	Cecal villous adenoma	Good recovery; NR
Ongom et al. (2013) [7]	*BMC Research Notes*	32, Female	Rectal prolapse, pain	Ileum → rectum	Right hemicolectomy	None	Good recovery; NR
McMahon et al. (2024) [8]	*ACS Case Reviews in Surgery*	78, Female	Anal bulge, constipation	Rectosigmoid → anal canal	Rectosigmoid resection + ileostomy	Rectosigmoid lead point; ischemic rectum	Ileostomy later reversed
Current case, 2025	*Present report*	68, Male	Diarrhea, abdominal pain, weight loss	Small bowel → sigmoid	Subtotal colectomy + end ileostomy	Poorly differentiated cecal adenocarcinoma	Stable; oncology follow-up ongoing

The extensive involvement necessitated a subtotal colectomy with end ileostomy rather than a segmental resection, as reduction was contraindicated given the strong suspicion for malignancy and the risk of tumor dissemination [1,2]. Histopathology confirmed a poorly differentiated cecal adenocarcinoma with weak neuroendocrine features, staged pT3N0M0 (Stage IIA, AJCC 8th edition). Poor differentiation and intermediate tumor budding are recognized high-risk features in Stage II disease [9]. While guidelines permit observation in Stage II colon cancer, the presence of such features often justifies consideration of adjuvant chemotherapy [10]. In this case, the oncology team discussed capecitabine, 5-fluorouracil/leucovorin, or FOLFOX versus close surveillance. Importantly, molecular tools such as ctDNA, MMR status, and MSI/TMB profiling were integrated into the decision-making process, reflecting advances in personalized oncology care [11,12]. The patient’s postoperative course was further complicated by high-output drains, electrolyte derangements, and severe malnutrition (BMI: 16.1 kg/m²), underscoring the importance of meticulous fluid and electrolyte management and nutritional support in colectomy patients, particularly those with new ileostomies. This case contributes to the limited literature on adult pan-colonic intussusception secondary to colorectal carcinoma, emphasizing the rarity of the presentation, the importance of oncologic surgical principles, and the role of modern molecular diagnostics in guiding adjuvant treatment decisions.

## Conclusions

This patient with massive pan-colonic intussusception secondary to poorly differentiated cecal adenocarcinoma underwent a successful subtotal colectomy with end ileostomy. The postoperative course was primarily unremarkable, and the patient was discharged with a functional ileostomy and scheduled follow-ups with oncology. Unfortunately, the patient was lost to follow-up after four weeks. This report adds to the limited literature on adult pan-colonic intussusception, with poorly differentiated cecal adenocarcinoma as the lead point. It emphasizes the importance of considering malignancy in adult intussusception, the role of subtotal colectomy in extensive disease, and the evolving use of molecular tools such as ctDNA in guiding adjuvant therapy decisions.
